# Identification and characterisation of novel SNP markers in Atlantic cod: Evidence for directional selection

**DOI:** 10.1186/1471-2156-9-18

**Published:** 2008-02-26

**Authors:** Thomas Moen, Ben Hayes, Frank Nilsen, Madjid Delghandi, Kjersti T Fjalestad, Svein-Erik Fevolden, Paul R Berg, Sigbjørn Lien

**Affiliations:** 1 AKVAFORSK – Institute of Aquaculture Research, Ås, Norway; 2 CIGENE – Centre of Integrative Genetics, Ås, Norway; 3 Institute of Marine Research, Bergen, Norway; 4 Department of Biology, University of Bergen, Bergen, Norway; 5 Norwegian Institute of Fisheries and Aquaculture Research, Tromsø, Norway; 6 Norwegian College of Fishery Science, University of Tromsø, Tromsø, Norway; 7 Norwegian University of Life Science, Ås, Norway

## Abstract

**Background:**

The Atlantic cod (*Gadus morhua*) is a groundfish of great economic value in fisheries and an emerging species in aquaculture. Genetic markers are needed to identify wild stocks in order to ensure sustainable management, and for marker-assisted selection and pedigree determination in aquaculture. Here, we report on the development and evaluation of a large number of Single Nucleotide Polymorphism (SNP) markers from the alignment of Expressed Sequence Tag (EST) sequences in Atlantic cod. We also present basic population parameters of the SNPs in samples of North-East Arctic cod and Norwegian coastal cod obtained from three different localities, and test for SNPs that may have been targeted by natural selection.

**Results:**

A total of 17,056 EST sequences were used to find 724 putative SNPs, from which 318 segregating SNPs were isolated. The SNPs were tested on Atlantic cod from four different sites, comprising both North-East Arctic cod (NEAC) and Norwegian coastal cod (NCC). The average heterozygosity of the SNPs was 0.25 and the average minor allele frequency was 0.18. *F*_*ST *_values were highly variable, with the majority of SNPs displaying very little differentiation while others had *F*_*ST *_values as high as 0.83. The *F*_*ST *_values of 29 SNPs were found to be larger than expected under a strictly neutral model, suggesting that these loci are, or have been, influenced by natural selection. For the majority of these outlier SNPs, allele frequencies in a northern sample of NCC were intermediate between allele frequencies in a southern sample of NCC and a sample of NEAC, indicating a cline in allele frequencies similar to that found at the Pantophysin I locus.

**Conclusion:**

The SNP markers presented here are powerful tools for future genetics work related to management and aquaculture. In particular, some SNPs exhibiting high levels of population divergence have potential to significantly enhance studies on the population structure of Atlantic cod.

## Background

The Atlantic cod (*Gadus morhua *L) is a well-known teleost inhabiting the Atlantic Ocean from North Carolina to Greenland in the west, and from the Bay of Biscay to the Arctic Ocean in the east. Atlantic cod supports a commercially important fishery [1]. However, in recent decades stocks have declined dramatically, particularly in the North-Western Atlantic (reviewed in [2]). The species is presently an attractive candidate for aquaculture, with commercial farming already taking place in Norway, Canada, and Scotland.

Genetic markers are imperative for proper management of cod and for cod aquaculture, as well as for ensuring a sustainable coexistence of wild and farmed stocks. There is a need for genetic markers for refining the stock structure and assigning fish to populations [3,4]. Today, management is primarily area-based and tends not to take into account the existence of genetically distinct stocks with different life history traits in a given area. In aquaculture, markers are needed for the mapping of genes or Quantitative Trait Loci (QTL) influencing commercially important traits, and for pedigree tracking or testing. Thus far the most widely used genetic markers for Atlantic cod have been limited numbers of allozymes [5], Restriction Fragment Length Polymorphisms (RFLPs) [6], and microsatellites [7-12].

The largest population of Atlantic cod at present is the North-East Arctic cod population (NEAC) [13], located in the Barents Sea area. Although the feeding grounds are off-shore in the Barents Sea, the NEAC spawns along the Norwegian coast, particularly in the Lofoten and Vesterålen region. Thus, NEAC may at times be found in areas which are also inhabited by the more stationary Norwegian coastal cod (NCC). A long-lasting controversy has been focused on whether NEAC and NCC are environmentally selected groups from the same gene pool, separate populations, or even siblings species [14-16]. Differences between NEAC and NCC has predominantly been found for blood type E [17-19], the haemoglobin (*Hb-I*) alleles [17-22], the pantophysin (*Pan *I) locus [14,15], and at some microsatellite loci [4,16]. Little or no genetic differences have been detected at most allozyme- [5,21,23] and microsatellite loci [4,16] or at the mitochondrial cytochrome *b *locus [24]. At the *Pan *I locus, NEAC predominantly have the *Pan *I^B ^allele (> 90%), whereas NCC have predominantly the *Pan *I^A ^allele [15].

Expressed Sequence Tag (EST) sequences are very useful resources in genomics, and can be used for the detection of Single Nucleotide Polymorphism (SNP) markers [25]. SNPs are by far the most abundant type of genetic polymorphism, and using SNPs is the only option for constructing very high-density marker maps and cost-efficient high-throughput genotyping. In the present study, we used EST sequences from Atlantic cod to identify SNPs that were subsequently assayed for allelic variation in 95 specimens of Atlantic cod (NEAC and NCC) sampled from the wild-caught base population of a breeding programme run by the Norwegian Institute of Fisheries and Aquaculture Research [26].

## Results and Discussion

### SNP detection

A total of 17,056 EST sequences were aligned and used to detect SNPs. The ESTs were 5' sequences representing eight different cDNA libraries with five to several hundred animals per library. The average sequence length was 508 ± 137 bp (SD). The alignment of ESTs produced 1186 contigs and 207 single sequences (Table 1). Seven hundred and twenty-four putative SNPs were found, distributed on 415 contigs. The contigs having candidate SNPs consisted of two to 11 EST sequences.

**Table 1:** Summary of contig assembly from ESTs

EST sequences	17,056
Putative transcripts	1,393
Singletons	207
Contigs	1,186
Contigs with:	
2 ESTs	559
3 ESTs	201
4–5 ESTs	136
6–10 ESTs	149
11–20 ESTs	96
>20 ESTs	45
Contigs with putative SNPs	415

### SNP validation

**Figure 1:**
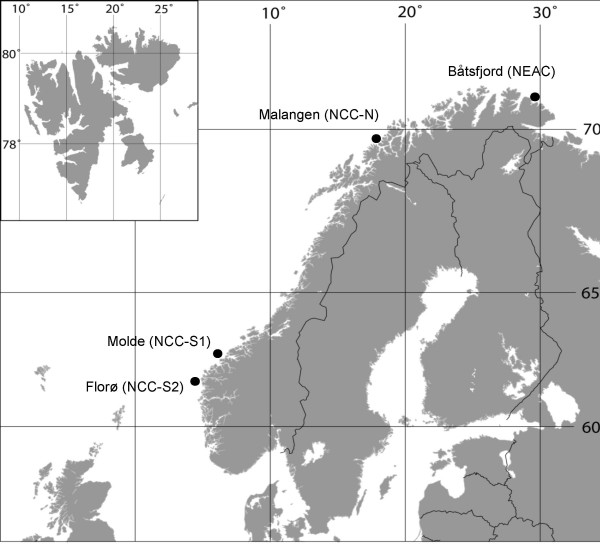
**Map showing sampling locations**. Sample abbreviations are described in Table 2.

Assays were successfully developed for 648 of the 724 putative SNPs. Five hundred and ninety-four of these 648 SNPs were genotyped on a collection of Atlantic cod sampled at four different locations; one site populated primarily by NEAC, one northern (NCC-N) and two southern (NCC-S1 and NCC-S2) sites populated primarily by NCC (Figure 1, Table 2). NCC-S1 and NCC-S2 were found not to be significantly different from one another (pairwise *F*_*ST *_= 0.013, p-value = 0.074), and are hereafter treated as one population, termed NCC-S. The animals that were genotyped had been preselected from larger samples based on their *Pan *I genotype. Thus, all NEAC were *Pan *I^BB ^and all NCC were *Pan *I^AA^.

**Table 2:** Samples used for SNP validation. All sampling locations were in Norway (Figure 1). The samples used for this study were selected from larger set of samples taken at the sites, selection being based on *Pan *I frequencies. Frequencies of *Pan *I^A ^among all fish sampled at the respective locations are given in column 3. *Pan *I genotypes of the animals used for SNP-genotyping are given in column 4. NEAC = North-East Arctic cod; NCC-N = Norwegian coastal cod north; NCC-S1 = Norwegian coastal cod south site 1; NCC-S2 = Norwegian coastal cod south site 2.

**Sampling location**	**Sample abbreviation**	**Freq. of Pan *I***^A^	**Pan *I *genotype**	**No of fish**
Båtsfjord	NEAC	0.125	BB	45
Malangen	NCC-N	0.872	AA	11
Molde	NCC-S1	0.966	AA	30
Florø/Nærøysund	NCC-S2	0.980	AA	9

**Figure 2:**
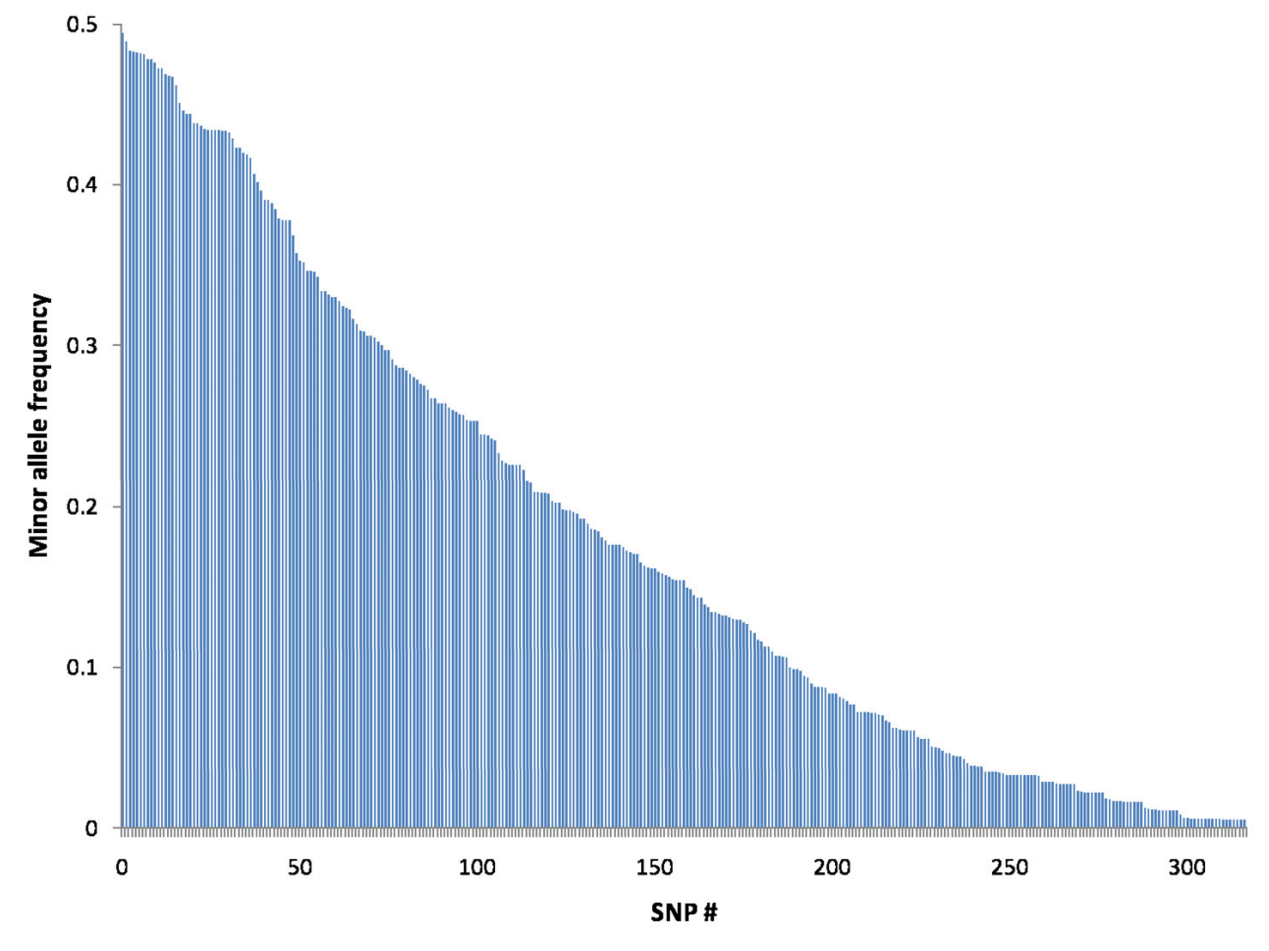
**Allele frequency distribution**. Overall allele frequency of SNPs, ranked according to allele frequency

Twenty-nine percent of the genotyped SNPs failed to amplify or did not cluster well according to genotype and were considered "failed assays", 2% were heterozygous in all animals and were thus assumed to represent duplicated genes, 15% were homozygous in all animals, while 54% were polymorphic and reliably scored (Table 3). The 318 polymorphic SNPs represented 235 contigs, with one to five SNPs per contig (Table 3). The mean minor allele frequency among the polymorphic SNPs was 0.18 ± 0.15 (SD) (Figure 2), while the mean overall heterozygosity was 0.25 ± 0.17 (SD). When homozygous SNPs were included, the heterozygosity was 0.19 ± 0.18 (SD). The true heterozygosity of real SNPs in the data set is likely to lie somewhere in between these two estimates, since we could not distinguish real SNPs that were homozygous in our material from loci that had falsely been identified as putative SNPs.

**Table 3:** Summary from validation of SNPs

Putative SNPs	724
Put. SNPs with assay successfully made	648
Putative SNPs genotyped	594
Failed assays	175
SNPs w/all animals heterozygous	9
SNPs w/all animals homozygous	92
Polymorphic SNPs	318
Contigs with putative SNPs	415
Contigs with real SNPs	235
Contigs with:	
1 real SNP	172
2 real SNPs	48
3 real SNPs	11
4 real SNPs	2
5 real SNPs	2

### SNP annotation

Annotation using BLASTN and BLASTX gave significant hits for all but 73 of the 594 genotyped SNPs. Synonymous/non-synonymous status of SNPs could be determined for 77 of 594 SNPs; the remaining 517 were either located outside coding regions, or the corresponding genes had not been annotated. The overall ratio of synonymous to non-synonymous SNPs was 0.44, which agrees well with results from other species. For example, Cargill et al. [27] and Lee et al. [28] reported the synonymous-to-total ratio to be 0.47 and 0.45 in humans and cattle, respectively. The annotations of those contigs that contained true SNPs are given in Additional file 1.

### Population assignment

Since animals had been assigned to populations based on genotypes at *Pan *I, we wanted to test for animals that might have been misclassified as NEAC, NCC-N, or NCC-S. The assignment test reassigned three animals in the NEAC sample to NCC-N, and one animal in NCC-N to NEAC. These four animals were culled from further analysis. The three misclassified animals from the NEAC sample had caused significant deviations from Hardy-Weinberg equilibrium (HWE) at individual markers with large *F*_*ST*_-values, indicating that a few high-*F*_*ST *_SNPs could be sufficient to substantially improve the accuracy of NEAC/NCC assignments compared to assignment with *Pan *I alone.

### Hardy-Weinberg equilibrium

After culling of misclassified animals, the fraction of polymorphic SNPs that were not in Hardy-Weinberg Equilibrium (HWE) (P < 0.05) was 0.10 for NEAC, 0.04 for NCC-N, and 0.09 for NCC-S, i.e. slightly higher than expected by chance. Four SNPs, Gm376_0668, Gm0927_0134, Gm0786_0527, and Gm392_1244, were out of HWE in all three populations, while 12 other SNPs were out of HWE in two populations [see Additional file 1]. Deviations from HWE could be due to genotyping errors or bias in the identities of uncalled genotypes. However, genotypes of SNPs out of HWE in one or more populations had been carefully double-checked, and the fraction of markers out of HWE were not changed significantly by removing markers with call rates < 0.90, indicating that genotyping error is not likely to be the only cause of deviations from HWE in the data set.

### Population comparisons

**Figure 3:**
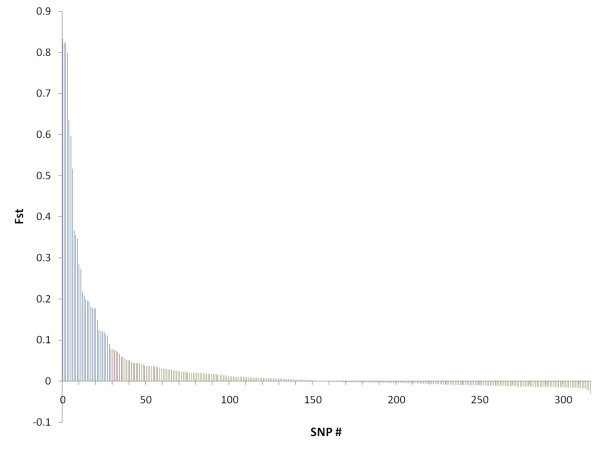
***F*_*ST *_values from locus-by-locus AMOVA**. *F*_*ST *_values were calculated at individual markers, with a three-population structure (North-East Artic cod (NCC), Norwegian coastal cod north (NCC-N), and Norwegian coastal cod south (NCC-S)). Blue columns = outlier loci according to the Beaumont and Nichols [29] test (Figure 4); red columns = non-outlier SNPs with *F*_*ST *_values significantly different from zero; green columns = SNPs with *F*_*ST *_values not significantly different from zero.

**Figure 4:**
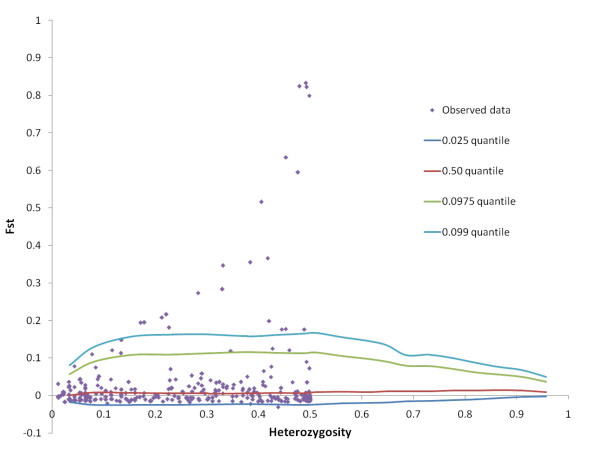
**Outlier SNPs**. The method of Beaumont and Nichols [29] was used to generate a distribution of *F*_*ST *_values versus heterozygosity under a neutral model. SNPs that had *F*_*ST *_values above the 0.975 quantile were considered outlier loci.

We wanted to investigate the potential of the SNP markers for discriminating different populations of Atlantic cod, in particular NEAC versus NCC, using the SNP genotypes described above. The mean pair-wise *F*_*ST*_-values were 0.057 and 0.024 for NEAC – NCC-N and NCC-N – NCC-S respectively, both significantly different from zero (P < 0.05). At individual markers, *F*_*ST*_-values ranged from 0 to 0.83 (Figure 3), and 48 SNPs had *F*_*ST*_-values significantly different from zero [see Additional file 1]. The Beaumont and Nichols test [29] revealed 29 outliers from the neutral-model *F*_*ST *_distribution (Figure 3, Figure 4). The majority of these 29 SNPs had an allele frequency cline reminiscent of that observed at the *Pan *I locus [see Additional file 2]. The outlying SNPs represented 26 genes, of which five corresponded to ribosomal proteins, three were involved in muscle contraction, two were involved in immune response, seven had other known functions, and nine had unknown or ambiguous functions [see Additional file 2].

We did not aim to thoroughly investigate the complicated issue of Atlantic cod population structure in this study, given the limited number of populations investigated and the pre-selection of individuals according to their *Pan *I genotype. However, since very large *F*_*ST*_-values were observed, it seems reasonable to conclude that outlier loci are, or have been, subject to diversifying selection, and that the gene flow between populations is minor; allele frequency divergence at the scale observed here would else not be likely to occur. Selection could be acting i) on the outlier SNPs themselves or on other SNPs within the same gene, or ii) on other loci in linkage disequilibrium with the outlier SNPs. Given the large number of genes in any higher organism, the even larger number of SNPs, and the limited number of markers tested in this study, selection is quite likely to be acting on genes in LD with the observed genes rather than on the observed genes themselves. Determination of LD levels in Atlantic cod will thus be essential to differentiate these two possibilities. If LD can be found across large genomics regions, the outlier genes/SNPs detected here are not likely to be under direct selection. However, if LD can be found across only minor genomic regions, selection is likely to be acting directly on the genes/SNPs themselves, in which case the data indicate that as much as 11% (26 out of 235) of genes in Atlantic cod are under positive selection.

## Conclusion

In this study, we have described the identification and characterisation of several hundred potential SNP markers from Atlantic cod, resulting in 318 SNPs that were polymorphic and reliably scored in a sample taken from three different sampling sites. The SNPs have the potential to significantly enhance the resolution of population structure in the Atlantic cod and provide more precise estimates of effective population sizes and rates of gene flow. Even from this preliminary study, it is clear that a significant fraction of loci in Atlantic cod display very large differentiation between North-East Arctic cod and Norwegian coastal cod, and thus are very likely to be affected by directional selection. The SNPs also represent a valuable resource for genetic mapping in Atlantic cod, and may be used for performing genome scans for quantitative trait loci (QTL) for phenotypic traits. Work is presently underway in our laboratory to construct a linkage map containing these SNPs and anchoring microsatellites.

## Methods

### Construction of cDNA libraries and sequencing of ESTs

EST sequences in Atlantic cod were obtained from eight different cDNA-libraries, made from tissues collected from the head kidney, intestine, liver, pyloric saeca, spleen (two libraries; stimulated and non-stimulated), and embryos (two libraries). For the embryo libraries, 500 different embryos were used, while for the other libraries, 15 animals were used in total. The animals used for EST sequencing were all Norwegian coastal cod. Samples from individual fish were stored in RNALater (Ambion) prior to use. Total RNA was extracted from each individual separately by the RNA Easy Mini Kit (Qiagen, Venlo, the Netherlands) and an equal amount of RNA from five different individuals was pooled. Enrichment for polyA RNA was conducted using the Oligotex mRNA Midi Kit from Qiagen. Nucleic acid quality was measure using Agilent 2100 Bioanalyzer (Agilent Technologies, Santa Clara, USA) and quantity was measure using Nanodrop ND-1000 (NanoDrop Technologies, Wilmington, USA). Directional cDNA libraries were constructed using 5 μg of polyA enriched RNA using the pBluescript II XR cDNA library construction kit from Stratagene (Cedar Creek, USA) according to the manufactures recommendations. The quality (size) (Bioanalyser) and quantity (Nanodrop) of the produced cDNA was assessed prior to ligation. XL10-gold cells were transformed and blue/white screened. Positive (white) colonies were randomly picked and grown overnight in 96 well plates. Plasmids were purified using the Montage Plasmid Miniprep kit (Millipore, Billerica, USA). Sequencing was conducted using BigDye chemistry and they were run on an ABI3700 sequencer from Applied Biosystems (Foster City, USA). Sequencing was done from the 5' end of transcripts.

### Construction of EST contigs and identification of putative SNPs

Base-calling from chromatograms was performed using the Phred program [30]. Vector (pCMV-PCR) was masked using Cross-Match (P. Green, unpublished; [31]). PolyA and polyT sequences were also masked using a custom made script, to avoid false clustering on these motifs. Clustering and contig assembly was performed with the Phrap program, (P. Green, unpublished). Phrap was run with the options "-trim_start 50 ~minmatch 50. The PolyBayes program [32] was used to detect putative SNPs in the sequence alignments, and give a probability of being a true SNP to each base substitution. The prior polymorphism rate for PolyBayes was 0.001 In addition, all contigs were manually inspected for SNPs using the program Consed [33].

### Annotation of SNPs

In order to annotate the SNP-containing contigs, BLASTX was run against the Protein data base, Swiss-Prot and non-redundant GenBank databases. A significant database hit was defined as having an expectation value (E-value) below 1.0 × 10^-10^. All sequences with a significant BLASTX hit in Swiss-Prot were annotated by annotation transfer, inferring similarity of function from sequence similarity by applying the Gene Ontology (GO) assignments for the UNIPROT database, produced by the GOA project of the European Bioinformatics Institute [34]. The gene_association goa_uniprot database of 07.07.2004 was used (see [35] for details) together with GO terms form the GO release of 06.08.2004 [36]. The sequences were annotated based on the single best hit in the Swiss-Prot database. BLASTN was used to annotate contigs against the nucleotide data base. Putative SNP functionality (e.g. resulting in a change in amino acid sequence or no change in amino acid sequence, non-synonymous or synonymous changes respectively) was predicted using the cSnper program [37].

### DNA sampling

The fish originated from randomly selected wild-caught Norwegian coastal cod (NCC) from southern and northern locations as well as from North-East Arctic cod (NEAC) (Table 2). All samples were collected autumn 2003. Genomic DNA was isolated from ethanol preserved fin clips in a GenoM-48 Robotic Workstation (Genovision, Oslo, Norway) using Magnetic Bead kits for purification and extraction of nucleic acids (Genovision) following the manufacturer's instructions. The fish were genotyped to characterise the fish as coastal cod or North-East Arctic cod based on their Pantophysin (*Pan *I genotypes [38]. The fish used in this study were a subset of the samples taken at the locations, selected based on their *Pan *I genotypes, where *Pan *I^AA ^were regarded as NCC and *Pan *I^BB ^were regarded as NEAC (Table 2). Pan I genotyping was done according to Stenvik et al. [38].

### Primer design and genotyping

Genotyping of SNPs was performed using the MassARRAY system from Sequenom (San Diego, USA). PCR-primers and extension-primers were designed using the software SpectroDESIGNER v3.0 (Sequenom). Multiplexes and primer sequences can be found in Additional file 1. Multiplexing levels were between 20 and 29. All SNP genotyping was performed according to the iPLEX protocol from Sequenom (available at [39]). For allele separation, the Sequenom MassARRAY™ Analyzer (Autoflex mass spectrometer) was used. Genotypes were assigned in real time [40] by the MassARRAY SpectroTYPER RT v3.4 software (Sequenom) based on the mass peaks present. All results were manually inspected, using the MassARRAY TyperAnalyzer v3.3 software (Sequenom). SNPs were classified as "failed assays" (meaning that the majority of genotypes could not be scored and/or the samples did not cluster well according to genotype), "SNPs w/all animals heterozygous", "SNPs w/all animals homozygous", or "polymorphic SNPs", based on this manual inspection. SNPs that were out of HWE in one or several populations were double-checked.

### Analysis of genotype data

Mean pairwise *F*_*ST *_values were calculated using the "pairwise *F*_*ST*_" function of Arlequin v3.11 [41]. *F*_*ST *_values at individual SNPs were calculated using the AMOVA function of the same program. Permutation testing with 1000 iterations was used to calculate p-values for mean and locus-by-locus *F*_*ST *_values. Arlequin v3.11 was also used for exact tests of HWE equilibrium (100 000 Monte Carlo iterations, 1000 dememorisation steps), and for assignment of individual genotypes to populations. Outlier SNPs were tested for using the method of Beaumont and Nichols [29], as implemented in the software package FDIST2 [42]. In FDIST2, average heterozygosity was first calculated using the *datacal *function. Simulations were then run using this average heterozygosity, 20 000 iterations, and assuming 10 demes, 3 populations, 40 individuals per sample, and a stepwise mutation model (the number of demes was increased until the average of simulated *F*_*ST *_values was approximately equal to the observed average *F*_*ST*_, as suggested in [29]). Simulated *F*_*ST *_values were plotted against heterozygosity to yield a distribution for *F*_*ST *_under a neutral model. SNPs with *F*_*ST *_values above the 0.975 quantile were considered to be outlier loci.

## Authors' contributions

TM inspected contigs for SNPs, coordinated SNP genotyping, did data analysis, and wrote a draft of the manuscript. BH did contig assembly, *in silico *SNP discovery, and annotations. FN performed the library construction and EST sequencing. MD provided and prepared DNA samples, and did Pan *I *genotyping of samples prior to the study. KTF provided DNA samples, took part in planning of the study, and was project leader. SEF provided DNA samples and guidance for the project. PRB did multiplexing of SNPs and took part in SNP genotyping. SL provided SNP genotyping facilities, and took part in SNP discovery. All authors took part in writing, and approved of, the manuscript.

## Supplementary Material

Additional file 1 SNP details. Properties of all 318 SNPs that were polymorphic, segregating, and could be reliably scored (SNP name, contig name, multiplex, contig sequence, SNP sequence, primer sequences, BLASTX and BLASTN results, synonymous/non-synonymous SNP, genotype counts, allele- and genotype frequencies, heterozygosities, *F*_*ST*_-values, tests for HWE) Click here for file

Additional file 2 Population parameters of outlier SNPs. Properties of high-*F*_*ST *_outlier SNPs (*F*_*ST*_-value, BLASTX hit, biological function, synonymous/non-synonymous SNP, allele frequencies, tests for HWE) Click here for file
