# Supplementary material for: Identification and characterisation of novel SNP markers in Atlantic cod: Evidence for directional selection
Source: BMC Genet. 2008 Feb 26;9:18. doi: 10.1186/1471-2156-9-18 (PMC2288615; doi:10.1186/1471-2156-9-18)
Supplement: Additional file 2 — Population parameters of outlier SNPs. Properties of high-FST outlier SNPs (FST-value, BLASTX hit, biological function, synonymous/non-synonymous SNP, allele frequencies, tests for HWE) [file 1471-2156-9-18-S2.doc]

**Population parameters of outlier SNPs**

Population parameters for outlier SNPs, having *F_ST_* values larger than expected under a neutral model (> 0.975 quantile). NEAC = North-East Arctic cod; NCC-N = Norwegian coastal cod, north, NCC-S = Norwegian coastal cod, south; Syn. = Synonymous SNP; Non-syn. = Non-synonymous SNP; HWE Hardy-Weinberg equilibrium; NA = not applicable. Significance levels of *F_ST_* values were found by permutation testing in Arlequin v3.11.

| **SNP** | ***F_ST_*** | **BLASTX hit** | **Biological function** | **Syn. or non-syn.** | **Allele frequencies** | | | **HWE test (p-values)** | | |
| --- | --- | --- | --- | --- | --- | --- | --- | --- | --- | --- |
|  |  |  |  |  | NEAC | NCC-N | NCC-S | NEAC | NCC-N | NCC-S |
| Gm366_0514 | 0.83^****^ | Creatine kinase M-type | Muscle contraction/ATP consumption | NA | 1.00 | 0.50 | 0.11 | - | 0.56 | 1.00 |
| Gm366_1022 | 0.82^****^ | Creatine kinase M-type | Muscle contraction/ATP consumption | NA | 1.00 | 0.50 | 0.11 | - | 0.57 | 1.00 |
| Gm366_1073 | 0.82^****^ | Creatine kinase M-type | Muscle contraction/ATP consumption | NA | 1.00 | 0.50 | 0.11 | - | 0.50 | 1.00 |
| Gm394_0364 | 0.80^****^ | 60S ribosomal protein L10a | Translation | NA | 0.06 | 0.50 | 0.90 | 1.00 | 1.00 | 0.10 |
| Gm0738_0160 | 0.63^****^ | Complement factor B precursor | Immune response | NA | 0.00 | 0.45 | 0.69 | - | 1.00 | 1.00 |
| Gm0738_0228 | 0.59^****^ | Complement factor B precursor | Immune response | NA | 0.76 | 0.22 | 0.09 | 0.00 | 0.34 | 0.26 |
| Gm335_0159 | 0.52^****^ | 60S ribosomal protein L3 | Translation | NA | 0.96 | 0.69 | 0.38 | 1.00 | 0.49 | 0.13 |
| Gm240_0209 | 0.37^****^ | 40 S ribosomal protein S21 | Translation | NA | 0.94 | 0.50 | 0.50 | 1.00 | 0.01 | 0.00 |
| Gm183_0303 | 0.36^****^ | No hits | - | NA | 0.04 | 0.50 | 0.44 | 1.00 | 0.01 | 0.00 |
| Gm1156_0573 | 0.35^****^ | No hits | - | NA | 0.01 | 0.20 | 0.42 | 1.00 | 0.31 | 0.01 |
| Gm270_0430 | 0.28^****^ | 60S Ribosomal protein L5 | Translation | Non-syn | 0.95 | 0.95 | 0.58 | 1.00 | 1.00 | 0.51 |
| Gm1108_0332 | 0.27^****^ | Chitinase precursor | Response to other organisms | NA | 0.99 | 0.85 | 0.65 | 1.00 | 1.00 | 0.31 |
| Gm0637_0143 | 0.22^***^ | Dolichyl-diphosphooligosaccharide-protein glycotransferase | Protein amino acid N-linked glycosylation | NA | 1.00 | 0.85 | 0.74 | - | 1.00 | 0.08 |
| Gm1339_0238 | 0.21^****^ | Sex hormone-binding globulin | Hormone transport | NA | 0.75 | 0.95 | 0.99 | 0.66 | 1.00 | 1.00 |
| Gm0493_0267 | 0.20^****^ | No hits | - | NA | 0.12 | 0.45 | 0.43 | 0.37 | 1.00 | 0.49 |
| Gm397_0436 | 0.20^****^ | Comitin (CABP1-related protein p24) | Actin-binding protein | NA | 1.00 | 0.94 | 0.78 | - | 1.00 | 0.16 |
| Gm0339_0044 | 0.19^****^ | No hits | - | NA | 1.00 | 1.00 | 0.76 | - | - | 0.29 |
| Gm0289_0495 | 0.18^**^ | No hits | - | Syn | 0.01 | 0.38 | 0.19 | 1.00 | 0.22 | 0.58 |
| Gm392_1244 | 0.18^*^ | Creatine kinase M-type | Muscle contraction/ATP consumption | NA | 0.50 | 0.71 | 0.83 | 0.00 | 0.02 | 0.00 |
| Gm349_1196 | 0.18^*^ | Guanidinoacetate N-methyltransferase | Creatine biosyntheis/muscle contraction | NA | 0.09 | 0.30 | 0.43 | 1.00 | 1.00 | 1.00 |
| Gm1154_0166 | 0.18^****^ | 60 S ribosomal protein L5 | Translation | NA | 0.26 | 0.35 | 0.62 | 0.23 | 1.00 | 0.74 |
| Gm1386_0216 | 0.15^****^ | Vitellogenin-2 precursor | Lipid transport | NA | 1.00 | 1.00 | 0.83 | - | - | 0.56 |
| Gm0286_0449 | 0.12^***^ | No hits | - | NA | 0.54 | 0.83 | 0.79 | 1.00 | 1.00 | 0.65 |
| Gm1106_0459 | 0.12^****^ | Trypsin precursor | Protease activity | NA | 1.00 | 1.00 | 0.86 | - | - | 0.57 |
| Gm0734_0313 | 0.12 | Cytosolic nonspecific dipeptidase | - | NA | 0.48 | 0.33 | 0.19 | 5x10^-5^ | 0.03 | 0.12 |
| Gm0269_0307 | 0.12^*^ | No hits | No hits | NA | 0.00 | 0.1 | 0.28 | - | 1.00 | 0.23 |
| Gm1331_0391 | 0.11^*^ | Heat shock 70 kD protein | Response to heat | Syn | 0.86 | 0.95 | 1.00 | 0.58 | 1.00 | - |
| Gm273_0221 | 0.11^*^ | Ubiquitin | Protein catabolism | Non-syn | 0.00 | 0.00 | 0.12 | - | - | 1.00 |
| Gm137_0034 | 0.08^*^ | No hits | - | NA | 0.98 | 0.85 | 1.00 | 1.00 | 0.16 | - |

^*^P < 0.05

^**^P < 0.01

^***^P < 0.001

^****^P < 0.0001
